# Knitted Structures Made of Antibacterial Fibers Intended for Protective Gloves

**DOI:** 10.3390/ma16237276

**Published:** 2023-11-22

**Authors:** Emilia Smiechowicz, Barbara Niekraszewicz, Magdalena Klonowska, Marta Strzelinska, Emilia Irzmanska, Natalia Litwicka

**Affiliations:** 1 Department of Mechanical Engineering, Informatics and Chemistry of Polymer Materials, Faculty of Material Technologies and Textile Design, Lodz University of Technology, Zeromskiego 116, 90-924 Lodz, Poland; barbara.niekraszewicz@p.lodz.pl (B.N.); m.strzelinska1@gmail.com (M.S.); 2 Institute of Material Science of Textiles and Polymer Composites, Faculty of Material Technologies and Textile Design, Lodz University of Technology, Zeromskiego 116, 90-924 Lodz, Poland; magdalena.klonowska@p.lodz.pl; 3 Department of Personal Protective Equipment, Central Institute for Labour Protection—National Research Institute (CIOP-PIB), Czerniakowska 16, 00-701 Warsaw, Poland; nalit@ciop.lodz.pl

**Keywords:** antibacterial lyocell fibers, nanosilica, immobilized silver nanoparticles, knitted structures, protective gloves

## Abstract

At a time of growing epidemic hazards caused by a very rapid evolution of dangerous pathogens, there is a pressing demand for bioactive textiles. Therefore, the development of high-quality knitted structures that could be used as bioactive protective materials has become a priority. This publication describes the fabrication of functional knitted structures from previously prepared antibacterial cellulose fibers containing nanosilica with immobilized silver nanoparticles. The structural and physical parameters of knitted fabrics made from them were studied with a view to their potential application in bioactive protective gloves. Tests of the basic structural and physical parameters of the knitted fabrics did not show that the nanomodifier applied in fibers significantly impacts the physical properties of the resulting fabrics. Moreover, water vapor permeability, cut resistance, and pH test results relevant to the functional and protective properties of interest and to user comfort showed that the obtained fabrics can be used in the production of bioactive protective gloves.

## 1. Introduction

The growing demand for comfortable, sanitary, and hygienic textile goods has created an urgent need for the development of antimicrobial textiles. With the advent of new technologies, the users’ increasing needs in terms of protective bioactive hygienic products can be met without endangering human health, safety, or the environment. The demand for antimicrobial fabrics in healthcare is becoming more pressing by the day. Microorganisms are known to have a long survival time in a variety of protective disposable sanitary fabrics in medical products such as splash gowns, masks, head and shoe covers, surgical drapes and gowns, gloves, surgical positioning kits, overalls, and laboratory coats. According to literature reports, protective clothing is critical in preventing the transmission of pathogenic bacteria and viruses, including COVID-19 [1,2]. Such clothing is typically cheap and disposable; it is usually made of polypropylene and polyester fibers and provides protection against pathogens. Protection against disease transmission for individual consumers, medical staff, and hospital patients can be further bolstered by bioactive products with antimicrobial properties. It can be noted that the consumers’ progressive attitudes towards hygiene and life quality have created an abruptly expanding market for antimicrobial textiles, which in turn has stimulated intensive research and development efforts. The size of the global market for such textiles looks very promising, with substantial growth potential. In 2020, it was estimated at USD 9.04 billion and was projected to grow to USD 9.45 billion in 2021 and USD 13.63 billion in 2028 at a CAGR of 5.2% during the 2021–2028 period [1,3,4].

Knitted fabrics and products account for a large proportion of innovative, bioactively modified textiles. Manufacturers offer various types of fibers with special properties for applications in knitted structures. A family of special polyamide fibers called Sensil is produced by the Israeli company Nilit [5,6]. They include New Sensil^®^ Biocare fibers—sustainable premium nylon fibers with increased biodegradability in seawater and landfills as compared to conventional nylon. Another interesting example is Meryl^®^ Skinlife fibers incorporating antibacterial silver microparticles in a polymeric matrix. Their manufacturer claims that their antibacterial properties are very long-lasting, with fiber performance being tested after 100 washes at 40 °C. Knitted products made of these fibers exhibit antibacterial activity and reduce unpleasant odors while maintaining the natural microflora balance of the skin. This type of fiber is used in sports shorts, as well as underwear and socks. The American company Invista™ is another example of a manufacturer of fibers with special properties, including Thermolite^®^ and Coolmax^®^. Also noteworthy are Outlast^®^ viscose fibers made from a natural polymer—cellulose—and patented by Outlast Technologies Inc. The Outlast^®^ technology was initially developed specifically for NASA. It uses phase change materials incorporated in the form of microcapsules called Thermocules™ enclosed in a polymeric matrix. The addition of Thermocules™ allows Outlast^®^ fibers to absorb, store, and release heat. As a result, a product made from these fibers regulates the user’s microclimate and provides physiological comfort. Outlast^®^ viscose fibers are used in the production of bedding, blankets, covers, and mattress protectors [7].

High-quality antibacterial knitted fabrics represent a wide area of research in the growing textile sector, especially at a time of increased exposure to pathogenic viruses and bacteria. Khude et al. [8] obtained polyester–cotton knitted fabrics with antibacterial properties. Polyester–cotton blends (50:50) and polyester fibers were modified with polyester–silver nanocomposite fibers in three weight proportions: 10, 20, and 30%. The factors that primarily contributed to the greater antibacterial activity of knitted fabrics were a higher proportion of nanosilver-modified polyester fibers, coarser yarns (lower yarn count), and denser fabric (higher machine gauge). Ramazan and Rajendran [9] developed an antibacterial knitted fabric loaded with silver ions using two modification methods, i.e., pad-dry-cure and spraying. They found a noticeable improvement in extensibility and elasticity in 100% PES fabrics after antibacterial treatment as well as decreased elongation in 100% viscose (VIS) fabrics in the machine direction, while tensile strength grew significantly. The authors observed similar results for 50%/50% PES/VIS knitted fabrics. After antibacterial treatment, the absorbency of all fabrics was noted to decrease significantly. In their patent application (US 10,577,726 B2), Tao et al. proposed a biodegradable antibacterial knitted fabric obtained by constructing a special three-layer cross-sectional structure. The advantage of the structure is that it does not contain any special antibacterial substances, and yet it exerts a durable antibacterial effect. Due to these factors, the production of such a fabric brings many benefits: in particular, it is characterized by cost efficiency, a facile production process, and, importantly, long-lasting antibacterial activity [10]. Mickevičienė and Treigienė [11] described the effect of antimicrobial treatment of PA and PES plain and plated knits on their basic parameters, i.e., structure, thickness, and air permeability. The authors used two antimicrobial materials: Isys AG and an organic-inorganic binder Isys MTX (CHT, Tübingen, Germany). It was demonstrated that when designing knitted products, it is important to predict the direction and rate of dimensional changes as these can affect physical properties such as air permeability. The antibacterial-treated fabrics were shown to change the parameters of their structure, thickness, and air permeability, which were more closely related to treatment conditions (temperature, solution treatment, mechanical action) than to the use of antimicrobial and sol-gel treatment. Yu and co-workers [12] developed and tested multifunctional shielding warp knitted fabrics to increase occupational safety in both the electromagnetic (EM) industry and hospitals. One of the goals of the presented research was to obtain multifunctional knitted fabrics that would also exhibit an antibacterial capacity as a prerequisite for products worn by users working in environments with poor indoor air quality. The tests showed that in addition to EM shielding, the obtained materials offer the extra benefit of excellent antibacterial activity against *E. coli* and *S. aureus* based on inhibition zone measurements. Asfand and Daukantienė [13] estimated the effects of using different fiber mixtures, knit patterns, fabric direction, technical side, and finishing treatments (dyeing, softening, and antibacterial finish) on the possibility of improving fabric functionality, including bending stiffness. Tests showed that dyeing and softening increased the surface density of the knitted fabric, both wale and course densities, and decreased fabric thickness. The antibacterial finish applied to softened knitted fabrics did not change the physical properties of the samples. Ivanauskas and co-workers [14] obtained antibacterial knitted fabrics modified with copper particles synthesized using copper sulfate as a precursor and ascorbic acid as a natural reducing agent. The authors examined samples of woolen and cotton knitted fabrics with copper particles that showed antibacterial activity against *E. coli* and *S. aureus*. Due to their sizable and long-lasting effects on pathogenic bacteria, the samples were deemed suitable for liners in reusable masks.

The recent viral epidemiological crises have led to a significant increase in demand for specific and effective bioactive products. Research literature is increasingly focused on topics involving antimicrobial and antiviral chemicals and textile products, including bioactive gloves [15,16]. In a hospital environment, the use of contaminated gloves on clean surfaces can potentially spread dangerous hospital pathogens. Frequently touched surfaces, such as doorknobs, railings, countertops, etc., serve as collection and storage areas for pathogens. As a result, patients are at risk due to cross-contamination, which can result in serious infections. The main and crucial purpose of the development and use of protective gloves is to protect healthcare workers, and ultimately hospital patients, from such hazards. Bioactive gloves are a relatively new technology, and many of their aspects should certainly be further explored to make them more effective [17].

At the same time, protective gloves with bioactive properties for hospital use must meet the requirements of the standards PN-EN 420+A1:2012 [18] and PN-EN 388+A1:2019-01 [19] in accordance with Regulation (EU) 2016/425 of the European Parliament and of the Council of 9 March 2016 on personal protective equipment and repealing Council Directive 89/686/EEC [20]. Additionally, it has been proposed that such gloves should be evaluated by means of methods developed for assessing the end of service life reflecting workplace conditions [21,22]. Given the innovative approach in designing bioactive protective gloves, the presented study aims to provide a reference for the industry. The current system of harmonized standards for testing protective gloves provides essential information about the protection level of new products and can be used for comparing different products when selecting them for specific work conditions. However, such information is not exhaustive as regards the actual level of protection provided by protective gloves throughout their service life. The proposed evaluation protocol for mechanical factors and the end of service life of protective gloves used for hygienic purposes addresses this issue and may serve as an approximate indication of how long such products can be used by workers [23].

The objective of this work was to fabricate a knitted structure that incorporates antibacterial fibers produced at a prior stage of our research, which were presented in one of our previous publications [24]. The ubiquitous threats related to rapidly developing, dangerous pathogens provided the motivation for seeking an optimal structure for a knitted fabric intended for bioactive protective gloves using antibacterial fibers containing nanosilica with immobilized silver nanoparticles. The designed and manufactured knitted fabrics, intended as materials for protective gloves, were subjected to laboratory tests in terms of their structural, protective, and functional parameters.

## 2. Materials and Methods

### 2.1. Materials

Knitted fabrics were made from selected antibacterial fibers that were fabricated and tested at the first stage of our research and described in our earlier publication. The applied cellulose fibers had been modified with nanosilica with immobilized silver nanoparticles by the *N*-methylmorpholine *N*-oxide (NMMO) method [24]. Unmodified cellulose fibers were used as a reference.

### 2.2. Fabrication of Knitted Structures from Selected Fibers

Left–right weave knitted fabrics were made from selected Lyocell fibers (F-500) [24] in the form of a bundle of 18 elementary fibers.

Knitted structures were made in three variants:

- K-001—knitted fabric made of unmodified cellulose fibers.
- K-002—knitted fabric made of cellulose fibers modified with nanosilica with immobilized silver nanoparticles in the amount of about 500 ppm (F-500), fabricated on a machine with a needle knock-over depth lower than in variant I (K-001).
- K-003—knitted fabric made of cellulose fibers modified with nanosilica with immobilized silver nanoparticles in the amount of about 500 ppm (F-500), fabricated on a machine with a needle knock-over depth equal to variant I (K-001).

All knitted fabric variants were made at the Department of Knitting Technologies and Textile Machines on a laboratory single circular knitting machine with a classical knitting zone.

The technical characteristics of the knitting machine were as follows:

- machine diameter (in inches) *φ* = 4″
- needle gauge E14
- number of needles I = 169
- revolutions per minute *n* = 50–200 min^−1^

### 2.3. Methods for Determining the Structural and Physical Parameters of Knitted Fabrics Made of Lyocell Fibers

#### 2.3.1. Course and Wale Density of Knitted Fabrics

The course and wale densities of the obtained knitted fabrics were determined on the basis of the standard PN-EN 14971:2007 [25]. Measurements were conducted using a magnifying glass with a graduated ruler placed on the knitted fabric in such a way that the two edges of the glass were parallel to the wales (when measuring wale density) or the courses (when measuring course density). Then, the number of loops was counted using a dissecting needle with an accuracy of half a loop. Measurements were taken in ten different places on the fabric face, for both course and wale density.

#### 2.3.2. Fabric Areal Density

The areal densities of the tested knitted fabrics were determined in accordance with the standard PN-P-04613 [26]. Ten 5 cm × 5 cm fabric samples were prepared and each of them was weighed on an analytical balance. The areal density of the fabric was calculated from the following formula:

(1) $$M_{p}\left(\frac{g}{m^{2}}\right)=400\times m_{p}$$

where *m_p_* is the weight of the 5 cm × 5 cm knitted sample [g] and

*Mp* is the fabric areal density [g/m^2^].

#### 2.3.3. Fabric Thickness

The thickness of the tested knitted fabrics was determined in accordance with the standard PN-EN ISO 5084:1999 [27]. It was measured as the distance between the reference plate on which the fabric was placed and a parallel presser foot, which exerted some pressure on the surface of the tested product. A mechanical thickness gauge with an area of about 20 cm^2^ was used at a pressure of 125 g. Ten measurements were performed for each fabric with an arithmetic mean calculated for each of them.

#### 2.3.4. Tensile Strength and Relative Elongation of Knitted Fabrics

Maximum tensile strength and relative elongation were determined using the strip method, in accordance with the standard PN-EN ISO 13934-1:2013-07 [28]. Two sets of test samples were prepared for each knitted fabric variant. Each set consisted of fabric samples with a width of 5 cm and a length of 10 cm. They were cut in such a way that their longitudinal direction was parallel to the wales and courses (ten samples for each direction). Measurements were carried out using a Hounsfield H50K-S strength tester at an extension rate of 100 mm/min.

### 2.4. Methods for Testing Protective Knitted Fabrics Affecting User Comfort in Protective Glove Applications

Due to the intended application of the produced fabric in protective gloves, laboratory tests were conducted to determine its basic parameters in accordance with the standards PN-EN 420+A1:2012 [18] and PN-EN 388+A1:2019-01 [19]. The scope of the conducted tests was defined by the material available to the research team, but it should be stressed that the selected test procedures enabled the assessment of fabrics for bioactive protective gloves.

#### 2.4.1. Water Vapor Absorption

Water vapor absorption by materials intended for protective gloves was evaluated in accordance with the standard PN-EN 420+A1:2012, point 6.4 [18]. The test determines the safe amount of absorbed water vapor per 1 cm^2^ of material. Three samples with a diameter of 85 mm were cut out from each fabric variant. They were weighed, placed between two flanges, and sealed within an apparatus for determining vapor absorption. After 8 h of exposure to water vapor, the fabric was weighed again.

Water vapor absorption was calculated according to the following formula:

(2) $$A=\frac{m_{a}-m_{b}}{S}$$

where the following holds:

*m_a_*—sample weight after the test [g];

*m_b_*—sample weight before the test [g];

*S*—area of the tested sample [cm^2^].

#### 2.4.2. Water Vapor Permeability

The test for water vapor permeability of materials intended for protective gloves was conducted in accordance with the standard PN-EN 420+A1:2012, point 6.3 [18]. The method involves indirect determination of the mass of water vapor that passes through the material (at constant air flow) and is absorbed by a desiccant, which enables air circulation in a closed container. The exposure time of an external part of the glove with a diameter of 34 mm to water vapor should be at least 7 h at a temperature of 20 ± 2 °C and a relative humidity of 65 ± 5%. Water vapor permeability was computed from the formula below:

(3) $$P=\frac{60\times {∆}_{m}\times 400}{\pi \times d^{2}\times t}$$

where the following holds:

Δ*m*—difference in the weight of the bottle with the sample before and after the test [mg];

*t*—time between measuring the two weights [min];

*d*—internal diameter of the bottle [mm].

#### 2.4.3. Water Absorption and Desorption Taking into Account the End of Service Life

The test was conducted according to an internally developed procedure for determining the end of service life of protective gloves [23] and the standard PN-EN 20344:2012, point 7.2 [29]. Water absorption was determined at the final stage of the test, and then a desorption test was conducted. The method involves weighing a sample that is in direct contact with the platform and with rollers through which water is delivered at a flow rate of 7.5 ± 2.5 mL/min. The test duration was 1 h. Water absorption was calculated as follows:

(4) $$W_{A}=\frac{m_{F}-m_{O}}{A}$$

where the following holds:

*W_A_*—water absorption [mg/cm^2^];

*m_O_*—initial sample weight [mg];

*m_F_*—final sample weight [mg];

*A*—sample area [cm^2^].

Desorption is determined pursuant to the following formula:

(5) $$W_{D}=\frac{m_{F}-m_{R}}{m_{F}-m_{O}}\times 100$$

where the following holds:

*W_D_*—water vapor desorption [%];

*m_O_*—initial sample weight [mg];

*m_F_*—final sample weight [mg];

*m_R_*—sample weight after reconditioning [g].

#### 2.4.4. Abrasion Resistance Taking into Account the End of Service Life

The test was conducted according to an internally developed procedure for determining the end of service life of protective gloves [23] and the standard PN-EN 20344:2012, point 6.3 [29]. The method evaluates the resistance of materials to abrasion cycles tracing a Lissajous figure resulting from simple harmonic movements performed at right angles to each other using a Martindale apparatus (James Heal, Sterling, VI, USA). The purpose of the test is to determine the number of abrasion strokes until abrasive wear. The test result was determined organoleptically, based on observations of changes on the surface of the tested sample. The determination of abrasion resistance for linings was conducted under dry and wet conditions (with the sample exposed to a strong stream of water in the latter case). The adopted method for testing abrasion resistance according to the end of service life procedure expands the scope of evaluation to include product assessment under wet and moist conditions.

#### 2.4.5. Cut Resistance

The test was conducted in accordance with the standard PN-EN 388+A1:2019-01, point 6.2 [19], determining resistance to cutting with a circular blade with a diameter of 45 mm and a thickness of 0.3 mm at a total cutting angle of 30°. The blade advances horizontally in reciprocating motion, making a full rotation opposite to its direction, under the prescribed load of 5 N. Each of the test sets consisted of two fabric samples measuring 6 × 10 cm, which were cut at an angle of 45° to the longitudinal direction of the fabric.

#### 2.4.6. Tear Resistance

Tear resistance was tested according to the standard PN-EN 388+A1:2019-01, point 6.4 [19]. The method measures the force required to propagate a tear in the material along its longer edge at the midpoint of a 5 × 10 cm sample. The test was conducted using a universal tester (INSTRON, Norwood, MA, USA) with a sample clamp depth of 20 mm and a jaw displacement rate of 100 ± 10 mm/min.

##### 2.4.7. pH of Aqueous Extracts

The pH of aqueous extracts from textile products was measured according to the standard PN-EN ISO 3071:2020-8 [30]. Extracts were prepared by weighing 2 g of cut fabric and adding 100 mL of 1 M aqueous solution of potassium chloride as the extractant, in two repetitions for each fabric. After 2 h of extraction at 60 rpm, the extracts were subjected to potentiometric measurements using a glass electrode.

## 3. Results and Discussion

### 3.1. Characteristics of Knitted Fabrics Made of Lyocell Fibers

Organoleptic Assessment of Knitted Samples

**Figure 1:**
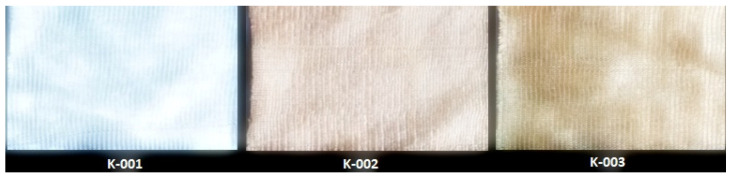
Images of all variants of knitted fabrics made of Lyocell fibers (K-001–K-003).

The obtained knitted fabrics exhibited different colors depending on the variant of the knitting process, and thus on the type of fibers used. Images of the fabricated products are shown in Figure 1. The structure of individual fabric variants was also imaged at an appropriate magnification using a Panasonic digital camera available at the Department of Knitting Technology and Textile Machines. Sample images are shown in Figure 1 and Table 1, Table 2 and Table 3.

Due to the intended use of the tested fibers for bioactive products, an important point in this study was to elucidate their antibacterial effects. An analysis of the results presented in our earlier publication [24] shows that F-250 fibers (250 ppm of silver) have no bactericidal activity against *Escherichia coli* and only negligible bacteriostatic activity. In turn, fibers with a higher nanomodifier content in the matrix (F-500, F-1000, and F-2000 fibers) exhibit strong bacteriostatic and bactericidal properties against *Escherichia coli* and *Staphylococcus aureus*. This means that the incorporation in the polymer matrix of silver nanoparticles immobilized on nanosilica in the amount of about 500 ppm in relation to α-cellulose is sufficient to obtain fibers with very good antibacterial activity against both gram-negative and gram-positive bacteria. Therefore, F-500 fibers (500 ppm of silver) were selected for microscopic analysis [24] as well as for the fabrication of knitted products. The resulting knitted structures are expected to exhibit antimicrobial properties comparable to the tested fibers.

The next research stage involved the fabrication of knitted products from the developed fibers. Weft-knitted fabrics with plain stitches were constructed using a single circular knitting machine at the Department of Knitting Technologies and Textile Machinery of the Lodz University of Technology. The fabrics were produced in three variants both from unmodified cellulose fibers and from fibers modified with nanosilica with immobilized silver nanoparticles in the amount of 500 ppm (F-500).

As can be seen from Figure 1, fabric K-001 (variant I) is white, similar to the unmodified Lyocell fibers from which it was made. Since fabrics K-002 (variant II) and K-003 (variant III) were made of cellulose fibers modified with nanosilica with immobilized silver nanoparticles, they are golden yellow due to the presence of the nanomodifier. The effect of nanoparticles on the color of the cellulose fiber was described in our previous publication [31]. An organoleptic evaluation of the produced knitted fabrics revealed that the structure of the variant K-002 was rougher compared to K-003.

### 3.2. Structural and Physical Parameters of Knitted Fabrics Made of Lyocell Fibers

#### 3.2.1. Determination of Course and Wale Densities of Knitted Fabrics

Course and wale densities of the obtained knitted fabrics were determined using the method described in the Section 2. All produced fabric variants were tested to enable comparative assessment of their course and wale densities. The results in the form of mean course and wale densities, standard deviations, and variation coefficients are given in Table 4 and Table 5.

Analysis of the wale density of the produced knitted fabrics (Table 4) showed that the values of this parameter remained similar for all the tested fabrics, regardless of the adopted knitting process. Course density was almost the same for fabrics K-001 and K-003 (88 and 87 courses per 10 cm, respectively), but was considerably higher for variant K-002 (114) as can be seen from Table 2 and Table 5. This is attributable to applying different needle knock-over depths in fabrics K-001 and K-003 vs. K-002.

#### 3.2.2. Determination of Fabric Thickness and Areal Density

Tests were performed for the knitted fabric made of unmodified Lyocell fibers, as well as for fabrics made of nanosilica-modified fibers with immobilized silver nanoparticles. Measurement results in the form of mean values of areal density and thickness, standard deviations, and variation coefficients of the tested parameters are given in Table 6 and Table 7.

Analysis of the results contained in Table 6 shows that fabric variants K-001 and K-003 are characterized by a similar areal density of approximately 50 g/m^2^, in contrast to variant K-002, which has a larger areal density of about 56 g/m^2^. This is due to its higher course density, resulting from a lower needle knock-over depth in the knitting process.

The data contained in Table 7, presenting the results of thickness measurements, indicate similar values for fabrics K-001 and K-002, amounting to 0.37 mm and 0.38 mm, respectively. The smallest thickness was found for variant K-003 (0.36 mm), which results from its smoother surface in organoleptic assessment. These properties may arise from the specific conditions of fiber making as well as from the fabric production technology.

#### 3.2.3. Determination of Tensile Strength and Relative Elongation of Knitted Fabrics

Tests were performed for all three variants of the produced fabrics: K-001, K-002, and K-003. Results are given in Table 8 and Table 9 as maximum force and tensile elongation values, standard deviations, and variation coefficients.

Maximum force measurements [N] along the wales and courses (Table 8) indicate that the values of the tested parameter for all fabric variants were much higher when the samples were stretched along the wales as compared to the courses. Fabrics K-001 and K-003 exhibited the highest maximum force of approx. 217 N and 210 N (along the wales), while the lowest value was recorded for K-002 (approx. 178 N). Analysis of this parameter for fabrics stretched along the courses revealed the highest value for fabric K-002 (approx. 45 N) and the lowest for K-001 (approx. 23 N). The parameter measured for fabric K-003 was about 30 N. For variant K-002, the maximum force was the highest among all the knitted fabrics stretched along the courses due to the largest number of loops per unit length in the direction of stretching (the highest course density).

Test results (Table 9) show that for all knitted fabric variants, relative elongation was significantly lower along the wales vs. along the courses. The smallest relative elongation along the wales was recorded for fabrics K-001 (approx. 20%) and K-003 (approx. 23%), and the highest for variant K-002 (approx. 39%). Analysis of this parameter along the courses reveals that relative elongation reached 382% for variant K-002, 398% for K-003 (the highest value), and 366% for K-001 (the lowest value).

### 3.3. Methods of Testing Protective Knitted Fabrics Affecting User Comfort for Applications in Protective Gloves

Due to the intended application of the fabricated knitted fabrics in bioactive protective gloves, an important aspect of this study was to determine the basic physical, protective, and functional parameters that have a direct bearing on the comfort and safety of individuals using the finished products (gloves) incorporating the fabrics. Consequently, we identified two basic parameters, namely, water vapor absorption and permeability, which define breathability and impact user comfort, according to PN-EN 420+A1:2012. Tests involved the bioactive knitted fabric K-003 made of cellulose fibers modified with nanosilica with immobilized silver nanoparticles in the amount of about 500 ppm (F-500). Since the fabric may affect user health and hygiene by remaining in direct contact with the skin, the pH of its aqueous extracts was determined. Its protective parameters were tested in terms of mechanical hazards in accordance with the standard PN-EN 388+A1:2019-01. The selected methods included the cut resistance test performed using the Couptest apparatus and the tear resistance test.

#### 3.3.1. Absorption, Desorption, and Permeability Properties of Knitted Fabric Intended for Protective Gloves

The absorption, desorption, and permeability properties of the knitted fabric made of modified cellulose fibers (Lyocell) were tested pursuant to the method described in the Section 2, with the results given in Table 10.

#### 3.3.2. Cut, Abrasion, and Tear Resistance of the Knitted Fabric Intended for Protective Gloves

The cut, tear, and abrasion resistance of fabric K-003 were tested pursuant to the methods described in Section 2.4, with the results given in Table 11.

#### 3.3.3. pH of Aqueous Extracts from Knitted Fabric Intended for Protective Gloves

The pH of aqueous extracts from the knitted fabric K-003 intended for protective gloves was determined pursuant to the method described in Section 2.4, with the results given in Table 12.

The knitted structure made from antibacterial fibers K-003 did not meet the normative requirements concerning water vapor absorption. At the same time, the sample satisfied the relevant standard in terms of high vapor permeability (100.4 mg/cm^2^·h).

While fabric K-003 failed to meet the standard concerning the recommended number of dry and wet abrasion cycles, the water absorbed by the sample was 100% desorbed, in accordance with the normative requirements.

Fabric K-003 exhibited low resistance to tearing, below the requirements of the standard. In turn, it reached performance level one in terms of cut resistance as defined in the standard.

The pH of aqueous extracts from the knitted fabric K-003 was tested with a view to its potential applications in the area of personal protective devices for the upper limbs and direct contact with the human skin. The obtained result was 5.8, which is well within the required range of 3.5–9.5.

## 4. Conclusions

Our study shows that the previously prepared fibers containing nanosilica with immobilized silver nanoparticles in the amount of 500 ppm (F-500) can be used to make a weft knitted fabric with a left–right weave.

Tests of the basic structural and physical parameters of the knitted fabric made of unmodified cellulose fibers (K-001) and the knitted fabric made of modified fibers (K-003), produced at the same needle knock-over depth, did not show any considerable differences, which implies that the nanomodifier used in the production of fibers had no significant effect on the physical properties of the resulting fabrics.

The methods of testing the basic physical and protective parameters of the obtained knitted fabrics relevant to the functional properties of interest and user comfort showed that fabric K-003 can be used in the production of bioactive protective gloves. This is supported by the fact that the properties crucial for the intended application, i.e., protecting the user against mechanical hazards and harmful substances, as reflected by parameters such as water vapor permeability, cut resistance, and pH, meet the requirements of respective standards.

Additionally, in previously conducted studies on the cytotoxicity of fibers, the authors concluded that the use of nanosilica is important from the point of view of limiting the release of nanoparticles and eliminating their negative impact on human cells [32]. Due to the high similarity of the research material (knitted structures made of antibacterial fibers modified with silver nanoparticles immobilized on nanosilica), the authors assume that the selected knitted fabric structure is also safe for human skin [32].

This means that the knitted fabrics made from fibers modified with nanosilica with immobilized silver nanoparticles could serve as functional textiles in the manufacture of antimicrobial protective gloves.

## Figures and Tables

**Table 1:** Structure of knitted fabric K-001 made of unmodified fibers.

Knitted Fabric K-001	
Back of the sample	Face of the sample
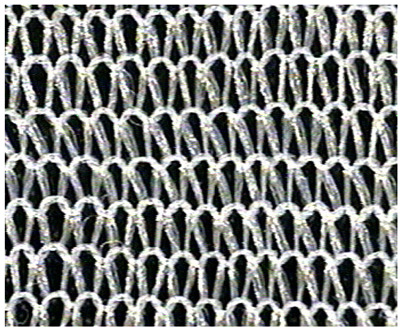	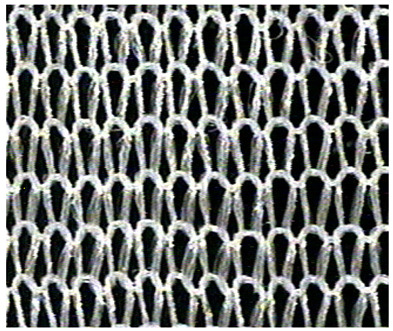

**Table 2:** Structure of knitted fabric K-002.

Knitted Fabric K-002	
Back of the sample	Face of the sample
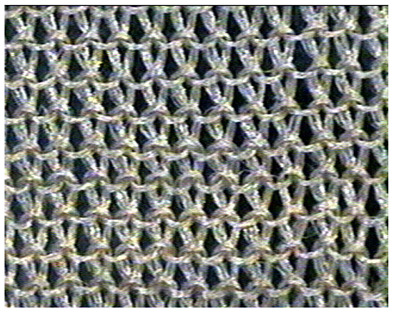	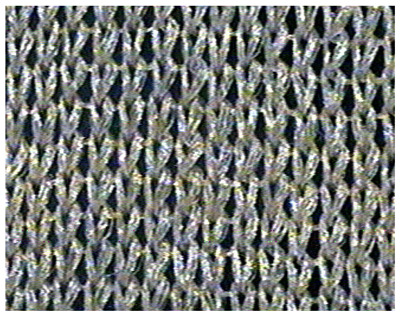

**Table 3:** Structure of knitted fabric K-003.

Knitted Fabric K-003	
Back of the sample	Face of the sample
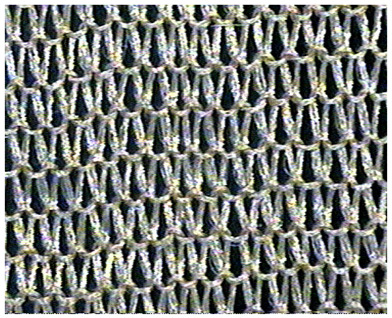	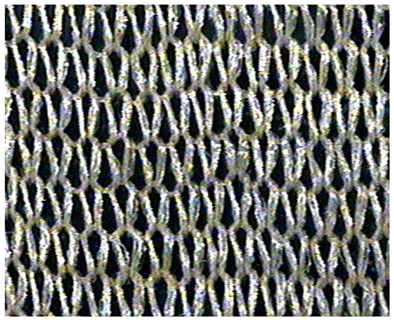

**Table 4:** Measurement results for the wale density of produced fabrics.

Sample Symbol	Wale Density [Number of Loops/10 cm]	Standard Deviation [Number of Loops/10 cm]	Variation Coefficient [%]
K-001	173	1.22	0.71
K-002	178	0.84	0.47
K-003	172	1.14	0.66

**Table 5:** Measurement results for the course density of produced fabrics.

Sample Symbol	Course Density [Number of Loops/10 cm]	Standard Deviation [Number of Loops/10 cm]	Variation Coefficient [%]
K-001	88	1.10	1.25
K-002	114	1.00	0.88
K-003	87	1.41	1.63

**Table 6:** Areal density results for produced fabrics.

Sample Symbol	Areal Density [g/m^2^]	Standard Deviation [g/m^2^]	Variation Coefficient [%]
K-001	49.32	3.00	6.08
K-002	56.38	2.46	4.36
K-003	50.82	1.90	3.73

**Table 7:** Thickness results for produced fabrics.

Sample Symbol	Thickness [mm]	Standard Deviation [mm]	Variation Coefficient [%]
K-001	0.37	0.01	2.25
K-002	0.38	0.01	2.19
K-003	0.35	0.01	1.58

**Table 8:** Maximum tensile force [N] along wales and courses for three produced fabric variants.

Sample Symbol	Maximum Force [N]		Standard Deviation [N]		Variation Coefficient [%]	
	Along Wales	Along Courses	Along Wales	Along Courses	Along Wales	Along Courses
K-001	216.95	22.95	16.05	1.84	0.07	0.08
K-002	177.70	44.78	11.74	1.31	0.07	0.03
K-003	209.55	29.75	12.37	3.89	0.06	0.13

**Table 9:** Relative elongation [%] along wales and courses for three produced fabric variants.

Sample Symbol	Relative Elongation [%]		Standard Deviation [%]		Variation Coefficient [%]	
	Along Wales	Along Courses	Along Wales	Along Courses	Along Wales	Along Courses
K-001	20.14	366.00	2.91	8.49	0.14	0.02
K-002	39.10	382.00	2.97	2.83	0.08	0.01
K-003	22.80	390.00	1.99	8.49	0.09	0.02

**Table 10:** Results of absorption, desorption, and permeability tests for the modified knitted fabric.

Fabric K-003	
Water vapor absorption by materials PN-EN 420+A1:2012, point 6.4	
Water vapor absorption 0.3 mg/cm^2^	Fails to meet the normative requirements Water vapor absorption during 8 h should be ≥8 mg/cm^2^
Water vapor permeability through materials PN-EN 420+A1:2012, point 6.3	
Water vapor permeability 100.4 mg/cm^2^·h	Meets the normative requirements Mean water vapor permeability should be ≥5 mg/cm^2^·h
Fabric K-003	
Water absorption and desorption According to the procedure for determining the end of service life	
Absorption 24 mg/cm^2^	Fails to meet the normative requirements Water absorption should be ≥70 mg/cm^2^
Desorption 100%	Meets the normative requirements Water desorption should be ≥80% of absorbed water

**Table 11:** Results of cut, tear, and abrasion resistance tests for fabric K-003.

Fabric K-003		
Cut resistance tests using the Couptest apparatus PN-EN 388+A1:2019-01, point 6.2		
Indicator I 1.2	Uncertainty ±0.1	Meets the normative requirements Fabric K-003 reached performance level one (at an indicator of I 1.2)
Tear resistance PN-EN 388+A1:2019-01 p. 6.4		
Tear resistance [N] 2 N	Uncertainty ±0.2	Fails to meet the normative requirements (performance level one at 10 N)
Fabric K-003		
Resistance of footwear lining to abrasion In accordance with the procedure for determining the end of service life		
Abrasion resistance [no. of cycles] Wet test (12,800–25,600)	Qualitative test	Meets the normative requirements Abrasion wear after 1000 cycles
Abrasion resistance [no. of cycles] Dry test (25,600–51,200)	Qualitative test	Meets the normative requirements Abrasion wear after 6500 cycles

**Table 12:** Test results for the pH of aqueous extracts from knitted fabric.

Fabric K-003	
The pH of aqueous extracts from knitted fabric PN-EN ISO 3071:2020-8	
pH 5.8	Normative requirements pH between 3.5 and <9.5

## Data Availability

Data are contained within the article.
